# Varicella-Zoster Virus Infection of Neurons Derived from Neural Stem Cells

**DOI:** 10.3390/v13030485

**Published:** 2021-03-15

**Authors:** Peter G. E. Kennedy, Trine H. Mogensen

**Affiliations:** 1 Institute of Infection, Immunity and Inflammation, College of Medical, Veterinary and Life Sciences, University of Glasgow, Garscube Campus, Glasgow G61 1QH, Scotland, UK; 2 Department of Infectious Diseases, Aarhus University Hospital, 8000 Aarhus, Denmark; trine.mogensen@biomed.au.dk; 3 Department of Biomedicine, Aarhus University, 8000 Aarhus, Denmark

**Keywords:** Varicella-Zoster virus, neural cell, stem cell, cell culture, latency, neuron, astrocyte

## Abstract

Varicella-Zoster virus (VZV) is a human herpesvirus that causes varicella (chickenpox) as a primary infection, and, following a variable period of ganglionic latency in neurons, it reactivates to cause herpes zoster (shingles). An analysis of VZV infection in cultures of neural cells, in particular when these have been obtained from induced pluripotent stem cells (iPSCs) or neural stem cells consisting of highly purified neuronal cultures, has revealed much data that may be of neurobiological significance. Early studies of VZV infection of mature cultured neural cells were mainly descriptive, but more recent studies in homogeneous neural stem cell cultures have used both neuronal cell markers and advanced molecular technology. Two general findings from such studies have been that (a) VZV infection of neurons is less severe, based on several criteria, than that observed in human fibroblasts, and (b) VZV infection of neurons does not lead to apoptosis in these cells in contrast to apoptosis observed in fibroblastic cells. Insights gained from such studies in human neural stem cells suggest that a less severe initial lytic infection in neurons, which are resistant to apoptosis, is likely to facilitate a pathological pathway to a latent state of the virus in human ganglia.

## 1. Introduction and VZV Biology

The pathogenic human herpesvirus Varicella-Zoster virus (VZV) is neurotropic, causing varicella (chickenpox) as a primary infection is unvaccinated children. The virus then remains in a latent state primarily in neuronal cells in several types of peripheral ganglia throughout the nervous system including the dorsal root ganglia (DRG), cranial nerve ganglia, particularly the trigeminal ganglia (TG), and also various autonomic ganglia including, as recently recognised, those located in the enteric nervous system [1,2,3]. Following a variable interval of a few years to many decades, the virus may subsequently reactivate to cause herpes zoster (shingles) which is a very painful cutaneous blistering and dermatomally distributed rash which in some cases can be followed by the excruciatingly painful condition known as post-herpetic neuralgia (PHN) which is typically chronic and refractory to most treatments. While herpes zoster may reactivate spontaneously, it may also occur after several different triggers such as malignant disease, immunosuppressant drugs, infections, and also increasing age [2,4]. It is now recognised that not only may VZV reactivation occur in the absence of the characteristic skin rash causing *zoster sine herpete* [5,6], but also such viral reactivation may also cause a wide variety of serious neurological complications. The latter have been described in detail elsewhere [3,7] and include encephalitis, myelitis, vasculopathy, segmental weakness and myelopathy, a variety of cranial neuropathies, Guillain-Barre syndrome, and gastro-enteric syndromes. There is also some evidence of the association of VZV with Giant Cell Arteritis [8] but this is a controversial issue and, as yet, not proven [3,9]. A detailed understanding of how VZV interacts with human neural cells is likely to enhance our understanding of the underlying pathogenesis of at least some of these VZV-associated neurological syndromes.

VZV has a double-stranded DNA genome the size of which is almost 125,000 base pairs and contains 68 open reading frames (ORF), three of which are duplicated [10]. The virus is very cell-associated and only infects human cells, in particular ganglionic neurons, T lymphocytes and epithelial cells [2]. Due to this strong cell association, it has been very difficult to obtain cell-free VZV in high titres, as opposed to the ease with which this can be achieved with herpes simplex virus (HSV) [11], and this problem has been a limiting factor in studying the VZV-neural cell interaction in vitro. VZV ganglionic latency has been studied in some detail but there are intrinsic difficulties in investigating this somewhat enigmatic process. Latent VZV DNA is known to be present in neurons in a non-integrated state, being either episomal or in concatemeric form [12]. It is certain that VZV gene transcription is limited during the latency process but the actual extent of this gene restriction remains somewhat controversial. One of the intrinsic problems with studying human ganglia obtained from autopsies is that the process of viral reactivation may already have started in ganglionic neurons very soon after death of the individual. Early studies reported the transcription of several VZV ORFs in human ganglia during latency including VZV ORFs 29, 21 62, 63 and 66 [13,14,15] but these studies examined ganglia removed up to 24 h after death raising the possibility that a degree of viral reactivation had already occurred, a possibility that was supported by a subsequent study that reported transcription of multiple ORFs including immediate–early, early and late genes transcribed in such ganglionic tissues [16]. More recent evidence suggests that when the ganglia are studied as early as less than 9 h after death, the only VZV ORF that can be detected is VZV ORF 63 (which has always been regarded as the hallmark of VZV latency) [14,17]. However, when the interval between death and human ganglia removal was reduced even further to about 6 h it was found that a novel unique spliced VZV transcript was detected in latently infected human TG neurons which was found to be mapped in an antisense configuration to the viral transactivator gene 61 [18]. Clearly VZV latency is a complex process that requires much further investigation.

In this overview we will consider some studies of acute and persistent VZV infection of human neural cells, focussing mainly on in vitro data. After considering some previous reports of viral infection of mature human and animal neural cells, we will then discuss some previous reports of VZV infection of neural stem cells. In practice these latter studies have mainly been in cell cultures obtained by differentiating induced human pluripotent stem cells (iPSCs), or embryonic stem cells into neurons, and these will be the main focus of this review.

## 2. VZV Infection of Cultured Neural Cells

Early studies of VZV infection of cultured human neural cells relied primarily on morphological methods of cell identification [19] as they predated the development of cell-type specific antigenic markers for unambiguously distinguishing the different types of human neural cells [20]. For instance, Gilden et al. [19] studied the replication of cell-free VZV in cultures of human brain and ganglion cells and compared viral growth to that in human fibroblasts. Descriptive assessments were made using a variety of methods including histology, electron microscopy (EM) and virological techniques. A multifocal cytopathic effect (cpe), including the presence of virus-specific intranuclear inclusions, developed in all cell types after 2–3 days and VZV infection of these cells was very cell-associated. While this study demonstrated that human neural cells were capable of being infected by VZV, cell-type specific markers to identify the different neural cell types were not available so the information gained was limited in this respect. A subsequent study infected fetal DRG cultures containing both neurons and non-neuronal cells with cell-associated VZV and did identify infected neurons using neuronal markers such as the A2B5 monoclonal antibody and the presence of neuron-specific enolase [21]. Despite the presence of VZV antigens (VZV glycoprotein) and a cpe observed in the identified neuronal cells, the latter were less susceptible to VZV-induced cytopathic damage than the supporting non-neuronal cells. This study had the disadvantage of not using cell-free virus but had the advantage of using double labelling immunofluorescence to identify virus-infected neurons. This differential susceptibility of neurons and non-neuronal cells to VZV infection has been confirmed in subsequent studies and may have relevance to VZV ganglionic latency.

A later study of heterogeneous populations of both central and peripheral neural cell cultures obtained from human fetal nervous tissue did used specific cell markers for both neurons and glial cells in culture. Thus Assouline et al. [22] used cell-free VZV to infect different heterogeneous human fetal cell cultures which contained neurons, astrocytes, Schwann cells and fibroblasts. Neurons were identified by their expression of A2B5, astrocytes by their cytoplasmic expression of glial acidic fibrillary protein (GFAP), and Schwann cells by both their characteristic morphology and the expression of the nerve growth factor (NGF) receptor. It was found that VZV replication occurred in all neural cell types 10–16 h after infection, but virus replication was slower in neurons and Schwann cells compared with that in astrocytes. Interestingly, there was relatively more VZV immediate–early protein in the perinuclear cytoplasm of neurons and Schwann cells than in other cell types suggesting the possibility of a delay in the transport of this VZV protein to its normal nuclear location. This study again showed the relative permissiveness of non-neuronal cells to VZV infection which could have pathogenic relevance, with astrocytes being the most permissive cell type.

The human astrocyte was also the main focus of a later study in which the cell free VZV OKA strain was used to infect dissociated cell cultures of human fetal brain [23]. The cultures contained 80% glial acidic fibrillary protein (GRAP)^+^ (positive) astrocytes and 20% GFAP^−^ (negative) fibroblastic cells. Using two-fluorochrome immunofluorescence, it was shown that both a cpe and viral antigen expression (VZV immediate–early protein 62 and gpI proteins) were detected in the cells a week after infection. However, it was found that astrocyte GFAP expression was both altered and diminished as the VZV infection progressed indicating a downregulation of GFAP expression by the virus itself. Detailed investigation showed that this downregulation was mediated by early rather than late events in the viral replication cycle and did not appear to be due to virally induced shut-off of host cell protein synthesis. These results were also observed using the multipassaged VZV Ellen strain, and the findings were thought to possibly represent a form of biological uncertainty phenomenon in that VZV itself was capable of modifying the result of the actual method of identifying the cell type that is was infecting. This finding of a VZV-mediated downregulation of GFAP was recently confirmed in an in vivo study of human VZV encephalitis [24].

A study also indicated that VZV may have unpredictable effects in a very different neural cell culture system which is felt to be relevant to the current discussion [25]. The aim of the study was to investigate whether VZV obtained from patients who had developed PHN following herpes zoster had different electrophysiological effects in a cell culture system compared with VZV that had been isolated from patients who had not developed PHN following zoster. VZV from both groups of patients were studied blind and were propagated as cell-free virus. The viruses were then exposed to the ND7/23-Nav1.8 rat DRG x mouse neuroblastoma hybrid cell line which showed constitutive expression of the exogenous Nav 1.8, and endogenous expression of Nav 1.6 and Nav 1.7 genes which encode sodium channels known to be abnormally regulated in different forms of neuropathic pain. Using the voltage clamp method, single cell sodium ion channel recording was performed in the neural cell cultures after 72 h. It was found that VZV associated with PHN increased significantly the sodium current amplitude in this cell line compared with the non-PHN VZV, and that these findings could be abolished by exposure of the infected cells to Tetrodotoxin (TTX). It was, therefore, concluded that the observed PHN-associated VZV sodium current increases were mediated at least in part, by the Nav 1.6 and Nav 1.7 sodium ion channels since these are known to be blocked by TTX. While intriguing, the relevance of these findings to the human neurological condition is unclear though there were clear differences in sodium channel modulation by the two groups of isolated VZV.

## 3. VZV Infection of Neural Stem Cells

There are at least four reasons why knowledge of the infection of neural stem cells may have potential importance in neurobiology and human neurological diseases. These are (a) to gain a better understanding of the biological basis of the VZV-neuron interaction using the various in vitro model systems, (b) they provide a mechanism of obtaining nearly pure terminally differentiated neuronal cultures which can be investigated for their susceptibility to viral infection, (c) they may give potential clues as to how VZV may interact with the developing fetal nervous system with clear relevance to congenital birth defects, and (d) they could provide a system in which to investigate the potential in vitro efficacy of therapeutic anti-viral agents. The discussion will include both VZV infection of embryonic neural stem cells and induced pluripotent stem cells

### 3.1. VZV Infection of Embryonic Neural Stem Cells

The intrinsic problems in studying VZV latency mentioned above inspired the search for new model systems, including neuronal stem cells, induced pluripotent stem cells (iPSCs) and neuronal organoid-like structures to make it possible to work with a human pathogenic virus like VZV in a relevant neuronal context in vitro. However, the number of existing neuronal stem cell derived and IPSC-derived neuronal models to study the virological and molecular aspects of VZV infection, replication and latency is relatively sparse, and these complex model systems remain challenging and often imperfect for mimicking true neuronal VZV infection and latency in humans.

To provide an additional way to study the VZV-host relationship in neurons, Pugazhenti and co-workers [26] developed an in vitro model of VZV infection of differentiated human neural stem cells. The neurons were maintained for up to 8 weeks and were infected with cell-free VZV (Zostavax vaccine) and expression of viral transcripts were compared to human fetal lung fibroblasts infected with VZV. An important observation which was consistent with previous results was that neurons were relatively resistant to apoptosis, whereas cpe and apoptosis were induced in fibroblasts [26]. In this kind of comparative study, it is important to expose the different cultures to an equivalent amount of virus, since the numbers of cells may be different and the multiplicity of infection (MOI) should always be the same.

Clearly, there are major differences between VZV infection of neuronal cells compared to other cell types, such as fibroblasts, which is also reflected in the differences between these tissues during the natural history of human infection with VZV, this being closely related to the ability of VZV to be neurotropic and establish neuronal latency. Several research groups independently have found that VZV-infected neurons remain viable for over 2 weeks, whereas fibroblasts tend to show cpe much earlier.

Although it has been demonstrated that neurons derived from pluripotent human embryonic stem cells (hESC) can be infected by VZV [27], it was subsequently realized that VZV did not replicate in naïve pluripotent hESC, implying that pluripotent hESC and neural progenitors at intermediate stages of differentiation are non-permissive for VZV infection [28]. Since this was found to be different from HSV infection, which was readily attainable in these more immature cells, the specific inability of VZV to infect more premature neuronal stem cells may provide important information regarding some of the characteristics of VZV neurotropism, latency, and intrinsic antiviral mechanisms in neurons. Another study reported that cell-free pOka and vOka elicited productive infection spread and release of infectious virus into the medium in hESC-derived neurons, and that the ability to establish a productive infection was highly dependent on the MOI, rather than on whether infection was performed with cell free or cell-associated VZV [29]. Importantly, such models may provide an opportunity for studying VZV reactivation and anterograde axonal transport of relevance to the pathogenesis of zoster.

One important unresolved question in the field and also complicating in vitro studies of VZV infection is related to VZV permissiveness in neurons and the unusual high level of viral cell association compared to most other herpesviruses. Using hESCderived neurons infected with cell-free wild-type VZV, it was demonstrated that neurons are highly permissive for VZV infection, and around 100 times more permissive compared to early-passage human embryonic lung cells or MRC-5 diploid human fibroblasts [30]. Specifically, the authors found that since VZV grows to much higher titers in human neurons than in other cell types, the number of total virus genomes relative to the number of viral particles that can form plaques in culture is much lower in human neurons than in other cultured cells. Interestingly it was also noted that neurons infected with VZV produced fewer defective or incomplete viral particles than fibroblasts (MRC-5 cells). This led the authors to suggest that hESC-derived neurons may be particularly useful for VZV propagation and also that such cells represent a more optimal cell type for studying VZV neuropathogenesis in vitro.

Some of the problems associated with working with terminally differentiated human neurons in vitro, i.e., their slow replication, relative short life span, and heterogeneity was addressed in a study by Goodwin et al. who described an experimental model of normal human neural progenitor cells in tissue-like assemblies or small three- dimensional organoids [31]. These organoids could be maintained for extended periods (at least 180 days) and could be infected with cell free VZV resulting in transcription of immediate early, early and late genes with an expression profile very similar to human trigeminal ganglia and with sporadic release of virus particles. Such models may represent physiologically relevant model systems and provide the potential for studying molecular mechanisms and interactions involved in long term VZV infection in these “complex assemblies” of human neuronal cells [31]. Although the model might be said to reflect permissive or persistent infection rather than true latency, one great advantage was the long term culture and lack of neuronal death under these experimental conditions. Collectively this system allows for studies of infection with VZV or other neurotropic viruses in a neuronal context with important similarities to human neuronal ganglia.

An important aspect that complicates and hampers studies and interpretation of VZV infection of in vitro-derived neurons is that these cultures are heterogenous containing cell types that may have different susceptibilities to VZV infection, establishment and maintenance of latency, and viral reactivation [32]. Therefore, a more precise and truer picture of VZV neuronal latency may be achieved when taking advantage of novel techniques to investigate these mechanisms at the single cell level [32]. Finally, some recent studies using of neurons generated from hESC were described in a study by Goldstein and colleagues who demonstrated the expression of bioinformatically predicted viral small non-coding RNAs, particularly microRNAs, during VZV infection [33,34]. These microRNAs are recognized as important actors in modulating gene expression and have thus been suggested to mediate an additional layer of regulation of the VZV life cycle and neuronal latency.

### 3.2. VZV Infection of Induced Pluripotent Stem Cells

There have been several published studies of acute VZV infection of induced pluripotent stem cells (iPSCs). These cells are derived from fibroblasts which have been experimentally dedifferentiated in vitro to mimic embryonic stem cells which are subsequently induced to differentiate into neuronal cells [35]. These studies have provided particular insights into the way in which VZV replicates in almost homogeneous neuronal cell cultures.

Lee et al. [36] devised a method to generate cell cultures of human sensory neurons from iPSCs which had been originally developed from human skin fibroblasts. After they had differentiated in culture the cells were characterised in several ways as sensory neurons. It was found that about 80% of the total cell population expressed the neuron-specific protein, βIII-tubulin, and 15% of the cell population co-expressed the markers Brn3a and peripherin, indicating that these cells were indeed sensory neurons. Their study showed that these sensory neurons could be infected by both VZV and HSV. Interestingly, it was found that cell-free VZV could infect human iPSC-derived sensory neurons and neural progenitor cells, but did not infect undifferentiated iPSCs.

Subsequent studies have analysed the VZV-neuronal cell interaction in detail. Grose et al. [37] generated highly pure (>95%) terminally differentiated neurons derived from iPSCs and infected them with cell-free VZV. Both viral transcription and ultrastructural features revealed by EM were investigated in these neuronal cells. It was reported that following infection VZV glycoprotein C (gC) was not expressed in most infected neurons and gC expression was markedly reduced in a minority of VZV-infected neurons whereas expression of the early–late VZV gE glycoprotein was abundant. Transcript analysis was consistent with these findings since there was also decreased gC transcription compared with gE. High resolution EM studies showed that in neurons there were fewer viral particles than would be typically observed in cells productively infected with VZV. The overall conclusion from these combined observations was that there was probably a significant deficiency in late-phase replication and viral assembly during VZV infection of the cultured neurons.

Another study carried out around the same time was that of Yu et al. [38] which infected highly pure (>95%) terminally differentiated human neurons derived from iPSCs with cell- free attenuated (vaccine strain) VZV. The neurons were confirmed as such in virtue of their positive labelling with the neuronal marker anti-βIII tubulin. Studying human fetal lung fibroblast cultures for comparison, these workers found that at 2 weeks post-infection, VZV- infected neurons appeared healthy compared to the infected fibroblasts which showed a cpe. Using molecular technology it was found that multiple regions of the VZV genome could be detected in these non-productively infected neurons, as could the full range of VZV transcripts and proteins. Interestingly, normal appearing infected neurons did not contain apoptotic protein markers.

Two further studies, both published in the same year, were reported from the Colorado research group which provided more insights into the growth of VZV in induced pluripotent stem cells containing almost homogenous (>95%) populations of neuronal cells. Thus, Baird et al. [39] studied further cell-free VZV infection in the same type of induced pluripotent stem cells as described and used in the above investigations. It was found that there was translation of VZV proteins from all kinetic classes with the production of only minimal infectious virus. It was also demonstrated that a low abundance of VZV DNA was correlated with the observed failure of the virus to produce neuronal cpe. Another study from this research group compared VZV-induced RNA transcripts in neurons and fibroblasts [40] in attempting to account for the lack of a cpe in VZV-infected neurons despite its presence in cultured fibroblasts derived from human fetal lung. Interestingly, analysis of detectable VZV transcripts in neurons and fibroblasts identified only 12 differentially transcribed genes of a total of 70 VZV ORFs, evidence that strongly suggested that defective virus transcription is not able to explain the observed lack of cell death of VZV-infected neurons in these cultures.

A recent study [41] also used infection of these highly pure neuronal cultures to further investigate whether VZV infection leads to neuronal apoptosis, since an earlier study [38] had suggested that it does not do so. Following infection with cell-free VZV it was found that VZV DNA did not accumulate in neurons and VZV transcripts in neurons were also only detectable at lower levels following viral infection when compared with human fetal lung fibroblasts. When protein markers of apoptosis were analysed it was reported that lower levels of the apoptotic marker cleaved Caspase-3 protein were detected in VZV-infected neurons compared with the human fibroblasts, and also higher levels of the known anti-apoptotic proteins Bcl2, Bcl-XL were found in VZV-infected neurons compared with uninfected cells.

## 4. Overall Synthesis

There are several key issues in the VZV-neural cell interaction which have been discussed here that remain to be better understood. First, why are non-neuronal cells such as glial cells more susceptible to acute lytic VZV infection than neurons? A clue may be found in the recent work described by Li Puma et al. [42] where astrocytes exhibited a higher susceptibility to HSV-1 infection than neurons, and this effect appeared to be dependent on a higher expression of the heparan sulfate proteoglycans, a binding site for HSV-1 located on the plasma membrane of astrocytes, and also the activation of a GSK-3-mediated pathway leading to virus entry and replication. Since HSV-1 and VZV share many of such features including a dependence on heparan sulphate proteoglycans expression and the GSK-3 pathway, it is conceivable that there may be a similar biochemical basis for the differential susceptibility to VZV observed in neuronal and glial cells. Future studies could focus further on these aspects. Second, why do undifferentiated human embryonic stem cells exhibit a lower degree of infection by VZV in comparison to HSV-1? Like Dukhovny et al. [28], we are unable to provide a definite explanation for this puzzling observation, but it may possibly be related to some kind of block to VZV infection in such cultures and/or the intrinsic nature of the usual cell-associated infection by VZV [28]. Since undifferentiated stem cells are generally rapidly dividing, it would be expected that viral replication in such cells would be greater than in post-mitotic cells. It is also of interest that undifferentiated stem cells may resemble glial cells such as radial glia [43], and that could partially explain the greater susceptibility of stem cells to HSV-1 infection. Third, why are neurons infected with VZV more resistant to apoptosis than non-neuronal cells? This may be explained in part by the increased neuronal expression of anti-apoptotic proteins and lower levels of the apoptotic marker cleaved Caspase-3 protein in VZV-infected neurons compared to non-neuronal cells [41]. While these may reflect the result of an initial process, it is possible that infected neurons are less susceptible to apoptotic signals designed to allow the infected host cell to terminate itself, thereby preventing viral spread. Fourth, what is the potential neurobiological significance of this neuronal resistance to virally induced apoptosis? These findings, with a less severe initial lytic infection in neurons, which are resistant to apoptosis, could potentially facilitate a pathological pathway to a latent state of VZV in human ganglia since they allow an ongoing viral infection to occur in viable neurons rather than an initial death of these cells which would terminate the viral infection.

Overall, many important aspects related to VZV tropism, replication, and latency in mature neurons, IPSC-derived neurons, and neural stem cells remain incompletely understood. Current and future studies should focus on addressing these questions using physiologically relevant models, such as freshly isolated post-mortem human sensory ganglia, together with investigations and comparisons of viral infection and cellular fate in neural progenitor cells, IPSC-derived neurons, astrocytes, and glia cells. Moreover, implementing experimental systems with neuronal three-dimensional cell assemblies or organoids should allow studies resembling the pathophysiological processes and immune responses occurring in human ganglia. Finally, genetic engineering by CRISPR/Cas9 technologies to modify or delete specific host genes involved in virus-cell interactions as well as using viral mutants should provide further insights into host-pathogen interactions in the human nervous system. Collectively, such approaches should provide deeper insights into the cellular and viral mechanisms governing VZV infection and establishment of latency in neurons with important parallels and relevance to VZV neuroinfection and disease in humans.

## Data Availability

Not applicable.
